# Suicide and Related Factors in Parkinson’s Disease

**DOI:** 10.1192/j.eurpsy.2022.2186

**Published:** 2022-09-01

**Authors:** G. Çakar, B. Duman, C. Akbostancı, H. Kumbasar

**Affiliations:** 1 Aksaray University Training And Research Hospital, Psychiatry, Aksaray, Turkey; 2 Ankara University Faculty of Medicine, Psychiatry, Ankara, Turkey; 3 Ankara University Faculty of Medicine, Neurology, Ankara, Turkey

**Keywords:** Depression, Suicide, hopelessness, Parkinson’s Disease

## Abstract

**Introduction:**

Parkinson’s disease(PD) is a progressive, neurodegenerative nervous system disease.Psychiatric symptoms are common in PD,it is important to consider the suicide risk of these patients.

**Objectives:**

The aim of this study is to investigate the risk of suicide in PD with a case control study and to determine the factors that may be associated.

**Methods:**

126 Parkinson’s patients and 117 age,gender matched healthy controls were included.Montreal Cognitive Assessment Scale(MoCA),Suicide Probability Scale(SPS),Beck Hopelessness Scale(BHS),Apathy Rating Scale(AES) and Multidimensional Scale of Perceived Social Support(MSPSS) were completed by participants.The PD group completed Parkinson’s Disease Quality of Life Questionnaire(PDQ-39) and data on disease duration,Movement Disorders Society-Unified Parkinson’s Disease Rating Scale(MDS-UPDRS),levodopa-equivalent daily doses(LEDD) were obtained.Hamilton Anxiety Rating Scale(HAM-A) and Hamilton Depression Rating Scale(HAM-D) were administered to all participants.

**Results:**

SPS total scores were significantly lower and total scores of HAM-A,HAM-D and MSPSS were found to be significantly higher in the PD group.There was no significant difference between the two groups in terms of BHS total scores.In PD group a linear relationship was found between SPS total scores and BHS,PDQ39, HAM-A and HAM-D and an inverse relationship with AES.In the regression analysis,it was concluded that a one-unit increase in BHS total scores increased the probability of suicide by 17.1%.

**Conclusions:**

It is seen that SPS scores in patients are lower than controls and observed that SPS is correlated with hopelessness,depression,anxiety and quality of life.Although the possibility of suicide is found to be low in PD,this risk increases in patients with untreated depression and anxiety.Therefore,psychiatric evaluation may be recommended in these patients.

**Disclosure:**

No significant relationships.

